# A Device for Prehabilitation of Total Knee Replacement Surgery (Slider): Usability Study

**DOI:** 10.2196/48055

**Published:** 2023-12-18

**Authors:** Riasat Islam, Daniel Gooch, Sudheer Karlakki, Blaine Price

**Affiliations:** 1 School of Computing and Communications The Open University Milton Keynes United Kingdom; 2 Robert Jones and Agnes Hunt Orthopaedic Hospital Oswestry United Kingdom

**Keywords:** physiotherapy, rehabilitation, prehabilitation, knee replacement, community physiotherapy, outpatient, gamification, motivation, adherence, knee, exercise, preoperative, postoperative, usability, validation, software, exergames, geriatric rehabilitation, self-regulated exercise, assistive technology, telerehabilitation, digital health, healthcare delivery

## Abstract

**Background:**

Rehabilitation, or “prehabilitation,” is essential in preparing for and recovering from knee replacement surgery. The recent demand for these services has surpassed available resources, a situation further strained by the COVID-19 pandemic, which has led to a pivot toward digital solutions such as web- or app-based videos and wearables. These solutions, however, face challenges with user engagement, calibration requirements, and skin contact issues. This study evaluated the practicality of a low-contact, gamified device designed to assist with prehabilitation exercises.

**Objective:**

The study aimed to assess the practicality and user-friendliness of a newly designed physiotherapy device (Slider) that enables exercise monitoring without the need for direct contact with the skin.

**Methods:**

A total of 17 patients awaiting knee replacement surgery at a UK National Health Service (NHS) hospital participated in this study. They used the device over a 2-week period and subsequently provided feedback through a usability and acceptability questionnaire.

**Results:**

The study was completed by all participants, with a majority (13/17, 76%) finding the device intuitive and easy to use. The majority of patients were satisfied with the device’s ability to meet their presurgery physiotherapy requirements (16/17, 94%) and expressed a willingness to continue using it (17/17, 100%). No safety issues or adverse effects were reported by the participants.

**Conclusions:**

The results indicate that the device was found to be a feasible option for patients to conduct presurgery physiotherapy exercises independently, away from a clinical setting. Further research involving a larger and more diverse group of participants is recommended to validate these findings more robustly.

## Introduction

In the United Kingdom, in the years 2019-2021, there were 237,934 primary knee replacements with 16,036 knee revision surgeries when the initial surgery was unsuccessful [1]. Patients must perform rehabilitation exercises before and after the operation to ensure proper healing and functioning of the joint. However, up to 70% of outpatients do not complete their physiotherapy, and 14% may not attend a follow-up outpatient appointment [2]. The strongest factor related to patient noncompliance is the various barriers that patients perceive and encounter in their treatment regimens. Another major reason for nonadherence is the lack of positive feedback that patients receive regarding the benefits of adhering to their treatments. Finally, the degree of helplessness that patients feel regarding their illnesses and treatments also substantially contributes to patients failing to comply with medical advice [3]. If the physiotherapy exercises are not completed, patients may be at an increased risk of revision surgery, thus increasing the cost and patient dissatisfaction [4].

Using an internet-enabled physiotherapy device at home can support a patient’s preoperative and postoperative knee replacement physiotherapy remotely, providing security and reassurance to both the clinician and the patient. Furthermore, this remote monitoring could free up clinician time and provide valuable objective data on the effects of physiotherapy on the quality and speed of recovery. By encouraging patients to complete their exercises, recovery can be optimized as patients are motivated to move the joint. Prehabilitation has been shown to augment postoperative function, improve recovery, and reduce hospital days [4-7]. It also serves as an educational tool to inform patients about the importance of performing these exercises postoperatively.

This study investigates the usability of Slider, a physiotherapy device intended to aid patients requiring knee replacements in their rehabilitation, developed by AI Rehab. The product was used according to its designated function in this study. The device is paired with a companion mobile app that tracks when patients perform their physiotherapy exercises and the frequency of completion. This data can be shared with the hospital to verify the accuracy of the exercises and monitor patient adherence to the program. It is used by placing it on a flat surface and having the patient push it along with their foot to ensure flexion and extension of the knee joint. The accompanying app incorporates all fundamental exercises relevant to total knee replacements. It records the manner in which exercises are performed, facilitating data sharing with the hospital for clinician review. This potentially reduces the time patients may wait for feedback from the clinic.

There are other products designed to facilitate at-home knee replacement physiotherapy. On one end of the spectrum, in terms of cost and simplicity, is Ortho-Glide by MYAID, allowing patients to slide their heel along the floor to facilitate knee joint motion [8]. This device does not incorporate sensors or patient guidance. On the opposite end of the spectrum, there is BPM Pathway, which uses a gyroscope and accelerometer attached to the patient's limb (usually the ankle) alongside an app that displays a real-time patient avatar and offers exercise guidance [9].

**Figure 1:**
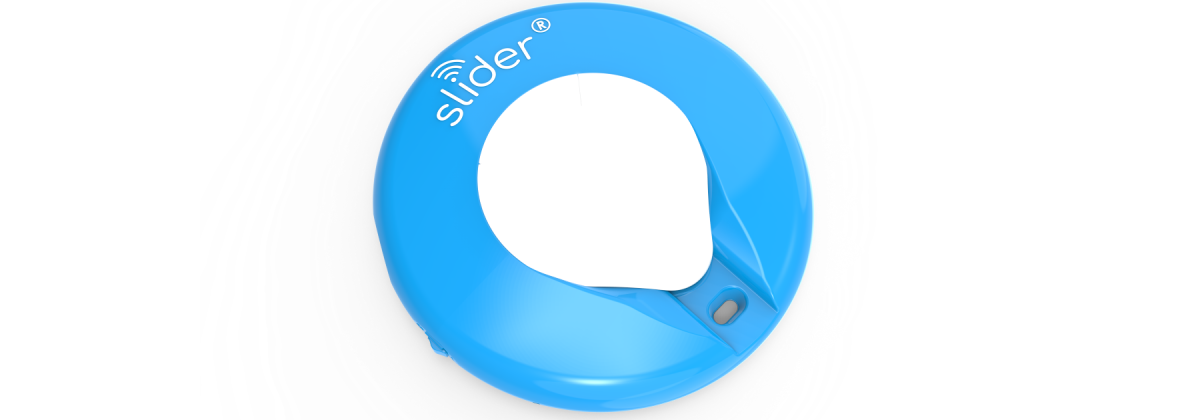
Slider device.

As shown in Figure 1, Slider is one such potential alternative. It aims to gamify exercises through an accompanying app while avoiding direct contact with the user’s skin. However, this device is a new product with little existing evidence about its usability in real-world conditions. This study investigates Slider’s preliminary viability as a prehabilitation solution. The focus is on evaluating the usability and acceptability of the device for physiotherapy exercises in a self-managed setting. While the device offers possible advantages such as simplicity and affordability compared to some other solutions, its actual usability by patients requires investigation. Therefore, the purpose of this study is to examine the following research question: “Does Slider provide a satisfactory user experience to complete the same physiotherapy exercises normally done in outpatient and hospital settings in self-managed free-living conditions?” This initial study will begin building evidence regarding the usability of the device. However, larger clinical studies are needed to further demonstrate the device’s outcomes and compare it objectively to alternatives.

## Methods

### Objectives and Outcome Measures or End Points

The primary objective is to establish the patient’s opinion on the usability and acceptability of Slider. The usability and acceptability internet-based questionnaire was completed after 2 weeks of using the device.

### Trial Design and Setting

A single-center study was run at The Robert Jones and Agnes Hunt Orthopaedic Hospital National Health Service (NHS) Foundation Trust in North West England.

### Ethical Considerations

The London City and East Research Ethics Committee approved the study (reference Integrated Research Application System [IRAS] 312158).

### Participant Eligibility Criteria

Participants were eligible for inclusion if they met all of the following criteria: (1) be on the waiting list for total knee replacement surgery, (2) are older than 45 years of age, (3) able to give informed consent, (4) able to speak and read English, (5) have active Wi-Fi internet at home, and (6) have access to an email address.

Participants were excluded if they had previous knee or hip replacement surgery on the same limb as their planned knee replacement surgery or if they had a stroke, a loss of sensation, or neuropathy affecting the side as their planned surgery.

### Trial Procedures

**Figure 2:**
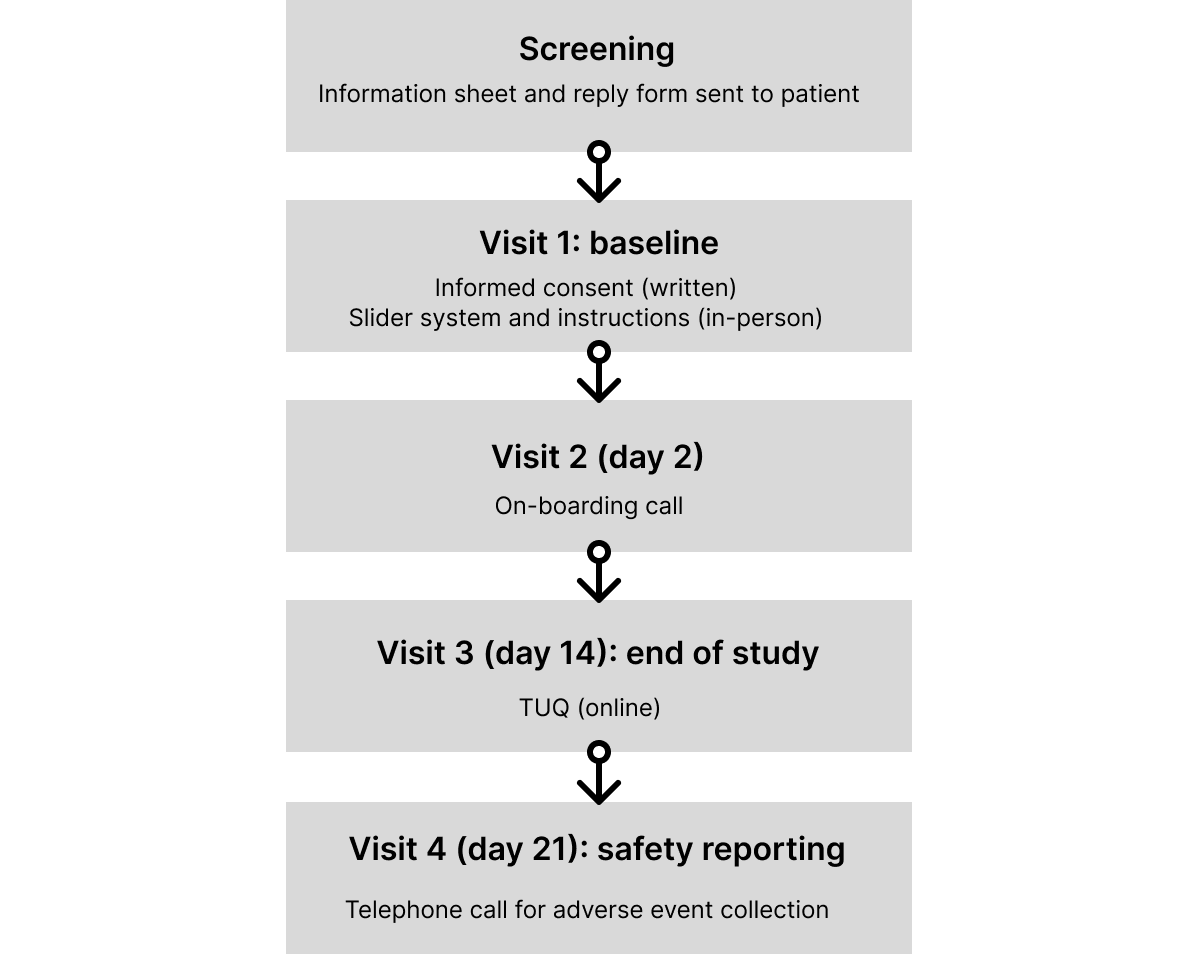
Schedule of events for the 2-week usability study. TUQ: Telehealth Usability Questionnaire.

The schedule of events is shown in Figure 2.

### Recruitment

#### Participant Identification

Potential patients were identified by reviewing the consultant waiting list for knee replacement surgery and the preoperative clinic lists. The lists were examined by the clinical team to identify individuals who met the eligibility criteria.

#### Payment

Participants were offered reasonable travel expenses for additional or research-specific clinical visits outside regular care.

#### Consent

Once identified, the patient information sheet and the reply form were sent to the patient. The returned reply form inferred consent for the patient to be approached about the study and to be contacted by a member of the research study team. A study team member contacted the participant by telephone to confirm consent for a study-specific appointment to receive the device and training. Specific permission was requested to share their contact details with AI Rehab to set up the software in preparation for receiving the device and consent to share details with the study team at The Open University. This enabled the completion of the poststudy internet-based usability questionnaire.

At the clinical visit for issuing the device and training, confirmed written consent was received from the participant by a research team member. Participants were reminded throughout that participation was entirely voluntary; that they were welcome to withdraw from the study at any time without recourse, redress, or comment; and that on request, their data would be destroyed any time up to the point of data aggregation, which will be within 5 working days from the date of final data collection, that is, completion of the poststudy questionnaire. Contact details were provided in the participant information sheet to expedite such requests.

#### Randomization

There was no randomization of the participants. All participants received a single device.

### Visit 1: Baseline

At the baseline visit, consent was confirmed, as described above. The exercises included in the Slider system are those usually prescribed for patients before and after knee replacement surgery. They feature in the standard treatment pathway, meaning the risk compared to usual care is similar. All participants were instructed on how to use the device by qualified physiotherapists, who are trained in the specific exercise program and prescribe exercise as part of their usual clinical practice. Each participant was required to use the device to perform 6 knee exercises twice daily for 14 days (2 weeks). The following exercises were encouraged at 5 repetitions per session, as shown in Table 1.

Each patient was provided with a Slider, an Android tablet device with the Slider app installed, a Slider mat, and charging equipment. The study team had access to the clinician-facing backend of Slider, where they could track the participants' progress. An instruction manual was provided with the system for the patients to take home.

**Table 1:** List of exercises and instructions on how to perform them.

Exercise	Demonstration	Instructions
Static quadriceps	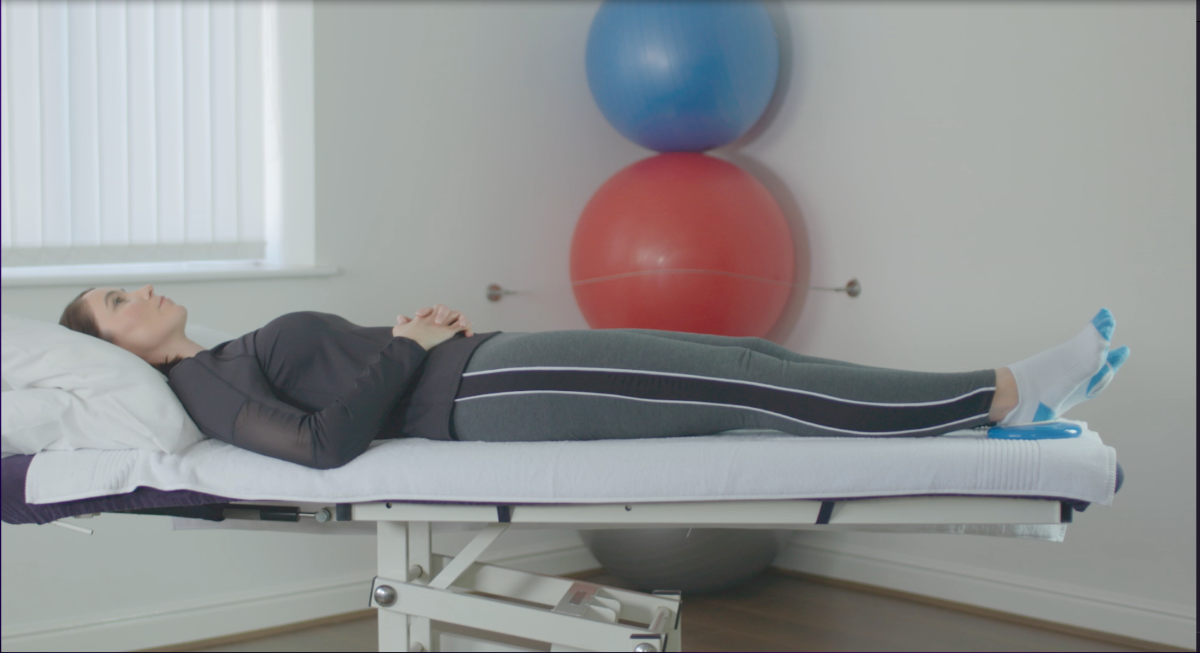	Lie on your back with legs straight. The Slider should be placed under your heel. Bend your ankle and push your knee down against the bed. Hold for 5 seconds and relax. Aim for up to 10 repetitions, but stop if fatigued.
Straight leg raise	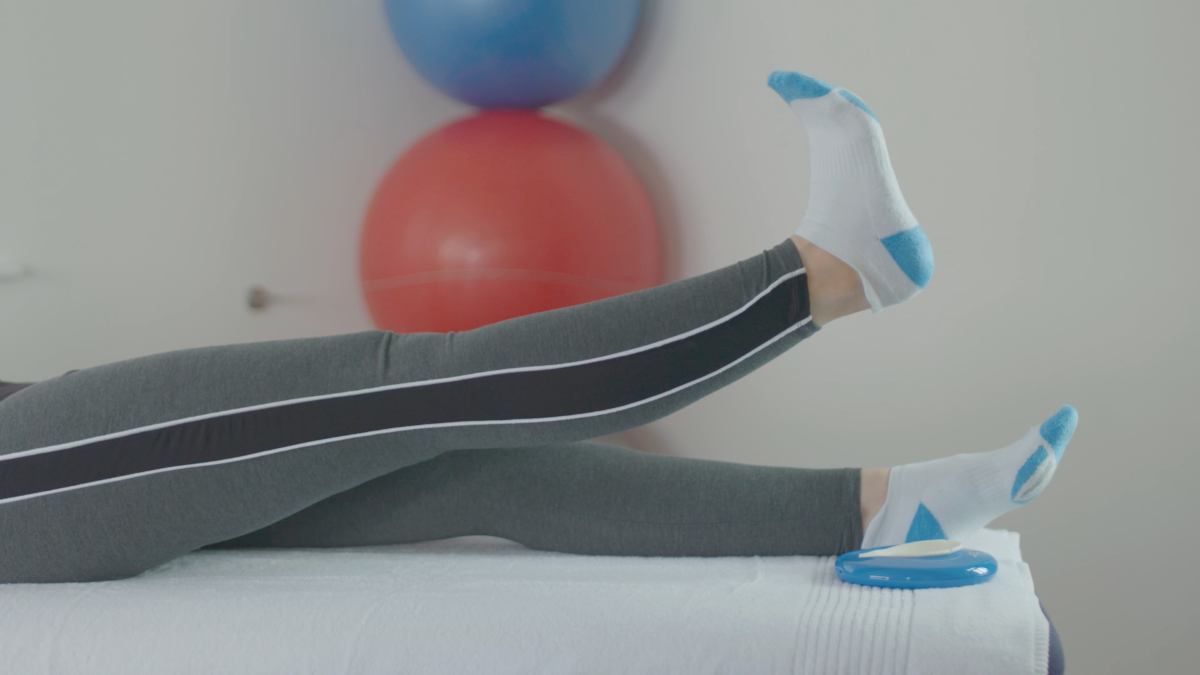	Lie on your back with your affected leg straight out and the other bent. The Slider should be placed under the heel of your affected leg. Exercise the straight leg by bending the ankle, contract the thigh muscle and lift the leg 20 cm or whatever is comfortable off the bed. Hold for 5 seconds. Aim for up to 10 repetitions but stop if fatigued.
Knee flexion lying	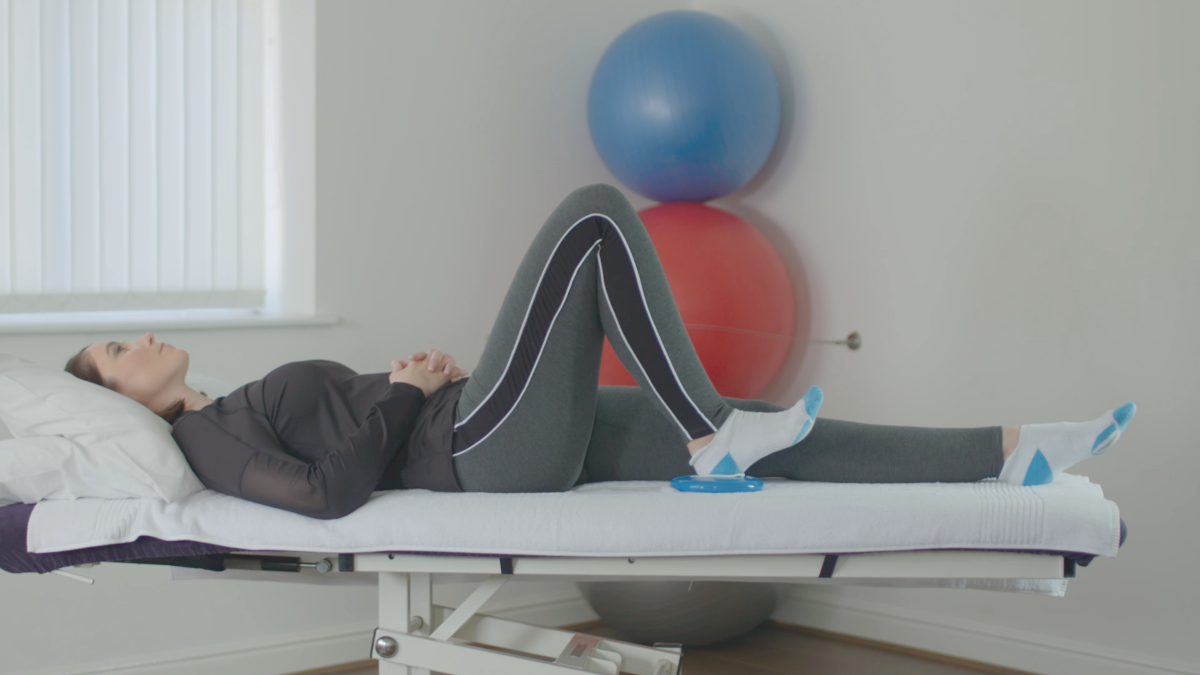	Lie on your back with your legs straight. The Slider should be placed under the heel of your affected leg. Bend your affected knee as far up as tolerable. Hold for 5 seconds then slowly lower your leg down into the straight position. Aim for up to 10 repetitions but stop if fatigued.
Inner range quadriceps	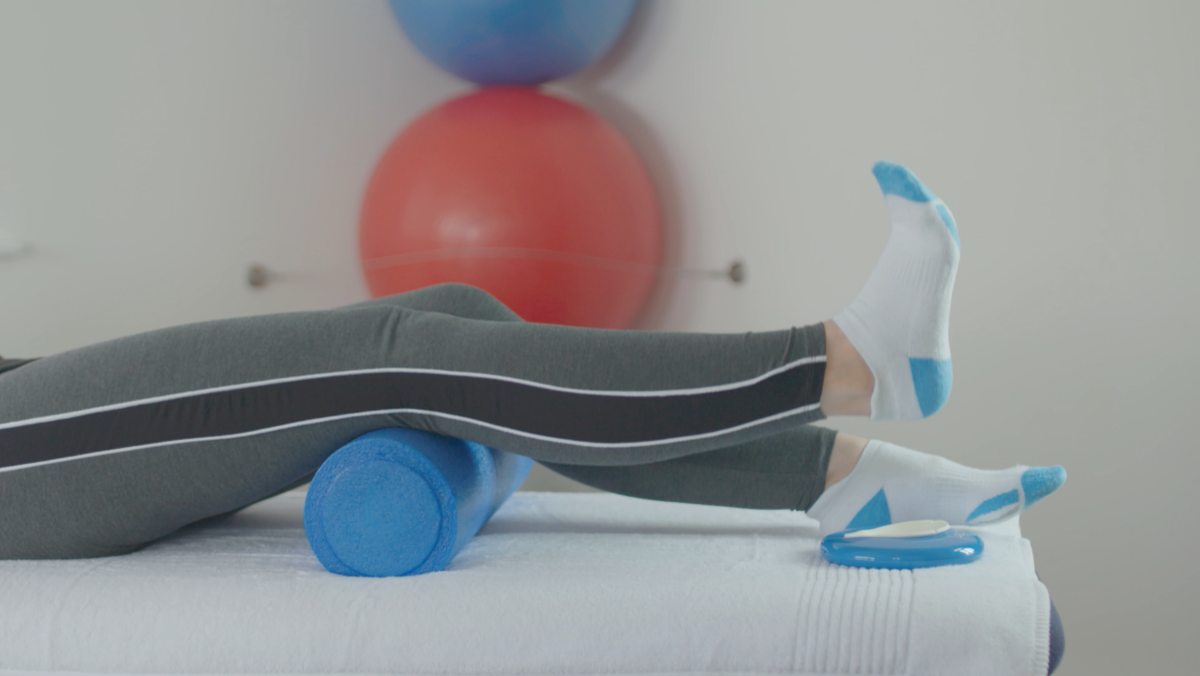	Lie on your back and place a rolled-up towel or pillow under your affected knee. The Slider should be placed under the heel of your affected leg. Bend your ankle and then straighten the knee making sure to keep the knee in contact with the pillow or towel. Hold for 5 seconds. Aim for up to 10 repetitions but stop if fatigued.
Knee flexion sitting	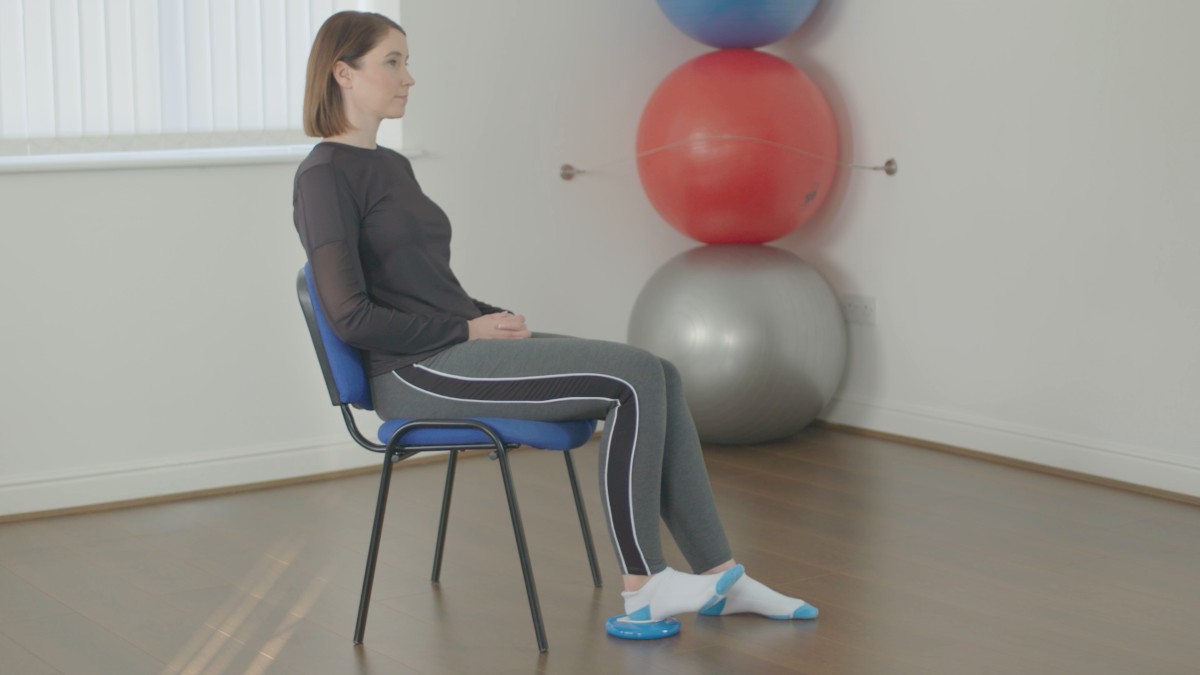	Sit on a chair with the Slider on the floor under the heel of your affected leg. Slide the foot under the chair as far as you can. Hold for 5 seconds and then straighten the leg keeping the heel in contact with the Slider. Aim for up to 10 repetitions but stop if fatigued.
Passive hyperextension	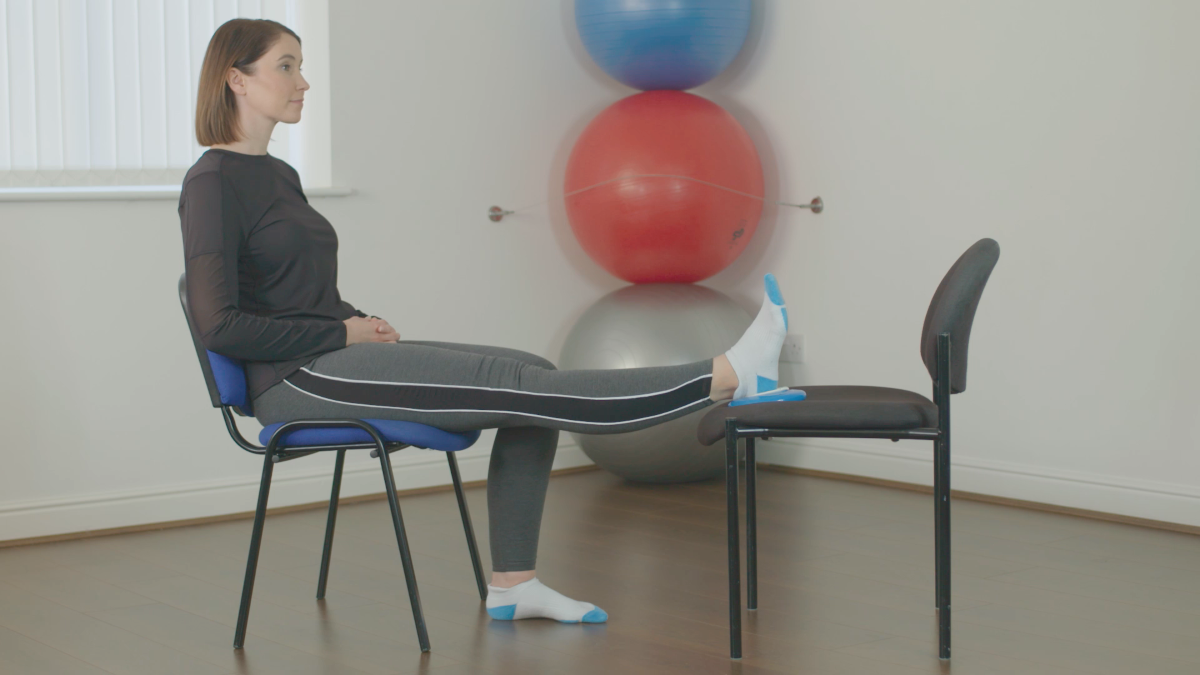	Sit on a chair with the affected leg supported on a stool and a Slider under the heel. Make sure there is no support under the knee to allow it to extend passively. Hold for 5-10 minutes as tolerated. Repeat 3 times a day.

### Visit 2: Day 2

On day 2, AI Rehab called the patients as part of their onboarding plan to ensure the system was set up correctly and to ascertain whether they required further support.

### Visit 3: Poststudy Questionnaire

Participants were asked to complete a poststudy internet-based usability questionnaire on day 14 of the study. The questionnaire requires approximately 10 minutes to complete and consists of generic demographic questions and an adapted version of the widely used and validated Telehealth Usability Questionnaire (TUQ) [10]. The responses were analyzed using descriptive statistics. The poststudy questionnaire is provided in Multimedia Appendix 1.

### Visit 4: Poststudy Serious Adverse Events Reporting

Participants were contacted by telephone by the study team within 3 days of the end of the trial period to record any adverse events during the 2-week exercise trial period, using the sponsor’s appropriate standard operating procedure—Clinical Trial Pharmacovigilance and Research Safety Reporting forms.

### End of Trial

The end of the study was defined as when 17 participants completed the 2-week trial of the device and the poststudy internet-based questionnaire. We provided participants with links to the resulting reports and outputs, allowing them to stay informed about the study's outcomes and results if they wished.

### Data Collection and Analysis

Once 17 participants completed their 2 weeks of using the device, they provided feedback via an internet-based questionnaire that assessed the overall system in 5 aspects: usefulness, ease of use and learnability, interface quality, reliability and satisfaction, and future use. Once all the responses were received, the anonymous data were aggregated, and the results were summarized. The results are reported in the *Results* section below with descriptive statistics.

## Results

This section presents the results from the usability assessment of Slider. Table 2 shows the demographic information of the participants. A total of 17 participants participated in the study. None of the participants discussed experiencing adverse effects or safety concerns while using this system. Among the participants, 3 (18%) people were between 55 and 64 years of age, 6 (35%) people were from 75 to 84 years of age, and 8 (47%) people were from 65 to 74 years of age. Regarding sex distribution, 10 (59%) people were female and 7 (41%) were male. A total of 10 (59%) participants performed exercises on their right side and 7 (41%) on their left side.

The TUQ provided the primary source of data for this study. TUQ measures user experience across 5 metrics, that is, usefulness, ease of use and learnability, interface quality, reliability, and satisfaction and future use.

**Figure 3:**
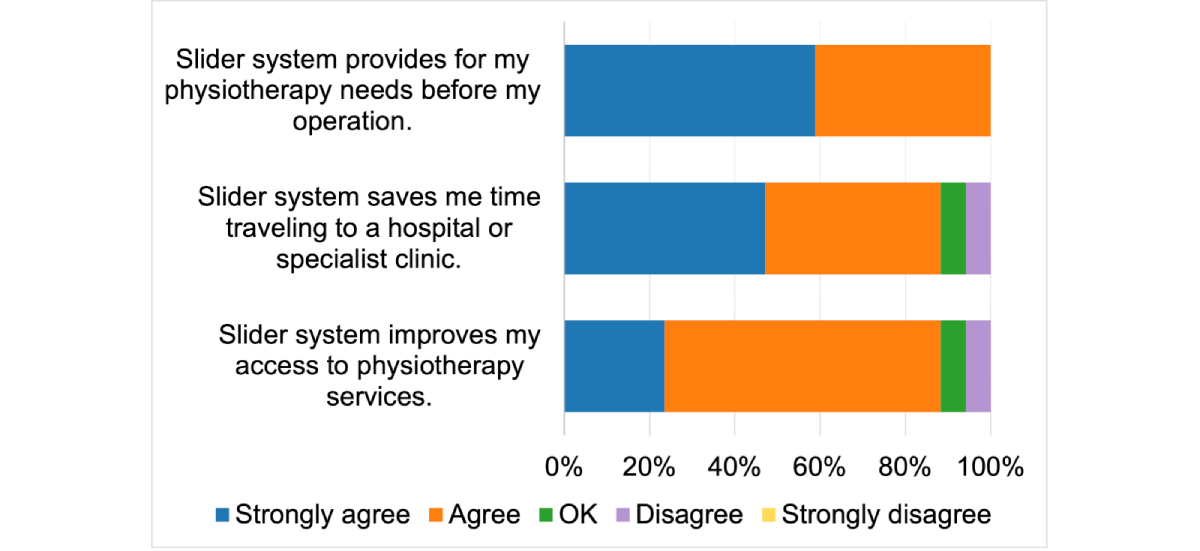
Telehealth Usability Questionnaire usefulness metric.

Figure 3 presents the results for TUQ’s usefulness metric. All participants agreed that the device provides for their preoperative physiotherapy needs. Regarding the statement about the device saving them time traveling to a clinic or hospital, a total of 15 (88%) participants agreed (8 participants strongly agreed and 7 participants agreed) with the statement, whereas 1 (6%) participant said OK, and 1 (6%) participant disagreed with the idea. A total of 15 (88%) participants (4 participants strongly agreed and 11 participants agreed) believed that the device improved their access to the physiotherapy system, 1 (6%) participant said OK, and 1 (6%) participant disagreed with the statement. Overall, 88% (15/17) of participants found the device useful for their preoperative needs.

**Figure 4:**
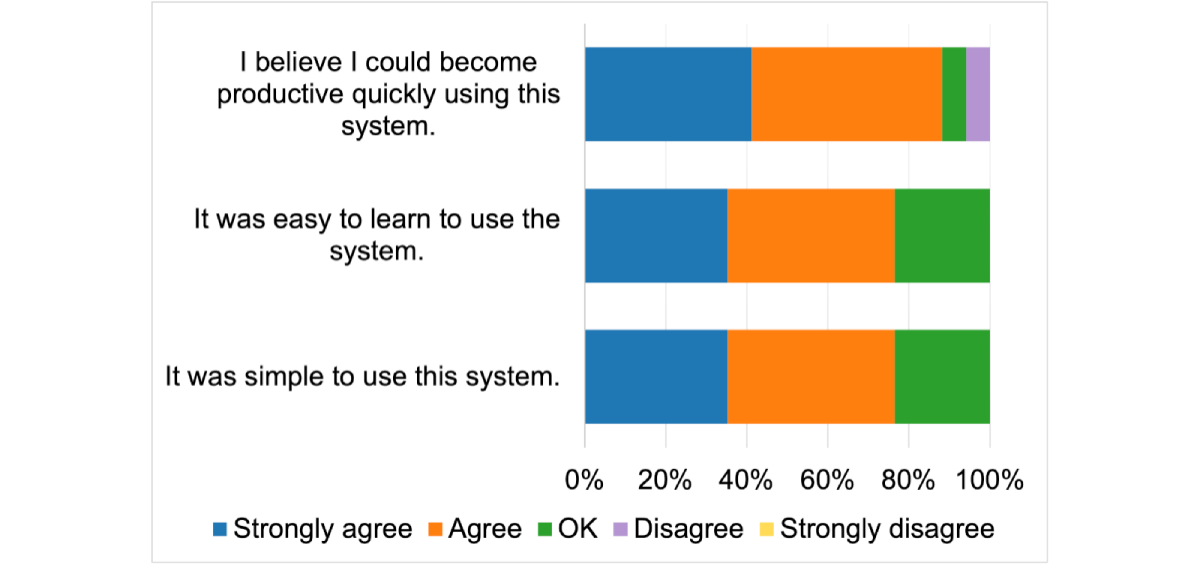
Telehealth Usability Questionnaire ease of use and learnability metric.

Figure 4 presents TUQ’s ease of use and learnability metric results. A total of 15 (88%) participants (7 participants strongly agreed and 8 participants agreed) believed that they could become productive quickly by using the device, 1 (6%) participant said OK, and 1 (6%) participant disagreed with the statement. When asked if the system was easy to learn, 13 (76%) participants agreed (6 strongly agreed and 7 agreed) with the statement. A total of 4 (24%) participants said OK and none disagreed with the statement. The same response was received when the users were asked if the system was simple. Overall, at least 13 (76%) participants found the device easy to use and learn.

**Figure 5:**
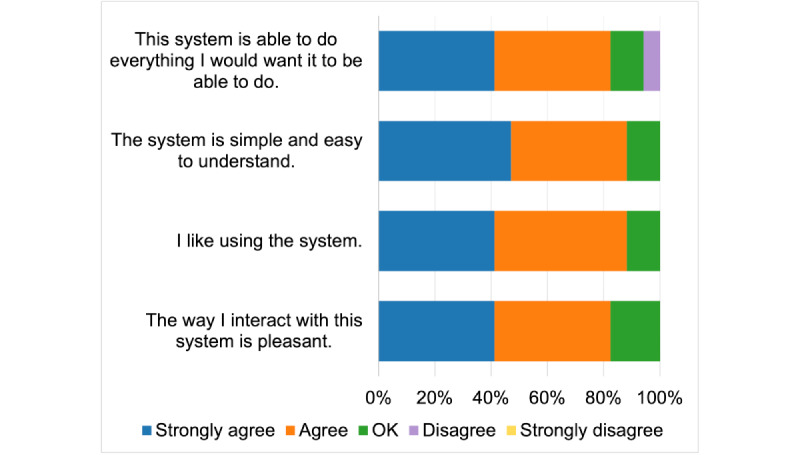
Telehealth Usability Questionnaire interface quality metric.

Figure 5 presents the results for TUQ’s interface quality metric. A total of 14 (82%) participants (7 strongly agreed and 7 agreed) found that the system could do everything it wanted. A total of 2 (12%) people stated that they found it OK and 1 (6%) person disagreed with the statement. A total of 15 (88%) participants (8 strongly agreed and 7 agreed) stated that the system was simple and easy to understand, whereas 2 (12%) people said OK and none disagreed with the statement. The same response was received when asked if they liked using the system. When asked if they found interacting with the system pleasant, a total of 14 (82%) participants (7 strongly agreed and 7 agreed) were positive. In contrast, 3 (18%) people said OK and none of the participants disagreed with the statement. Overall, at least 14 (82%) participants were pleased with the interface quality of the device.

**Figure 6:**
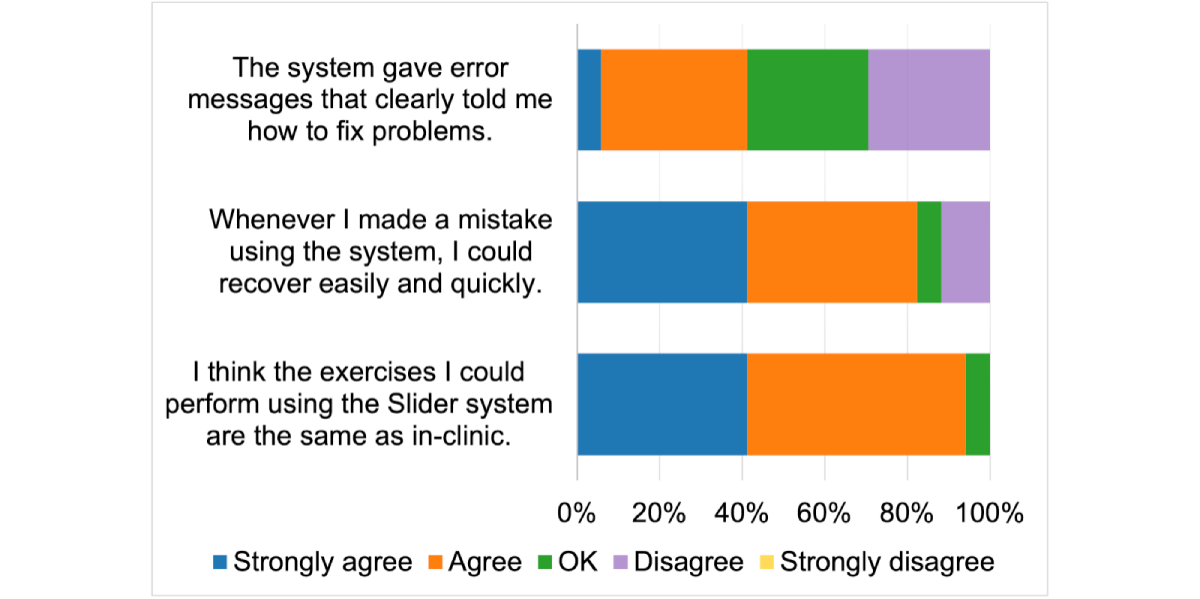
Telehealth Usability Questionnaire reliability metric.

Figure 6 presents the results for TUQ’s reliability metric. A total of 7 (41%) participants (1 participant strongly agreed and 6 participants agreed) said that the device gave error messages that told them how to fix problems. A total of 5 (29%) participants said they were OK with the statement, 5 (29%) participants disagreed, and none strongly disagreed. This question was ambiguous and likely caused confusion among the respondents. It is possible that some of the participants misunderstood this question and chose “disagree” because they did not receive error messages and therefore disagreed with “The system gave error messages...” rather than disagreeing that any error messages that may have occurred told them how to fix problems. However, as it was included in the submission for approval of the study, it was decided not to exclude it from the final report.

When asked if they made a mistake using the system and if they could recover easily and quickly, 14 (82%) participants (7 participants strongly agreed and 7 participants agreed) agreed with the statement. A total of 1 (6%) participant said they were OK and 2 (12%) participants disagreed. None of the participants strongly disagreed with the statement.

A total of 16 (94%) participants (7 participants strongly agreed and 9 agreed) agreed with the statement that the exercises they performed using the device were the same as in the clinic. All but 1 (6%) participant said they were OK with the statement and none of them disagreed with the statement.

**Figure 7:**
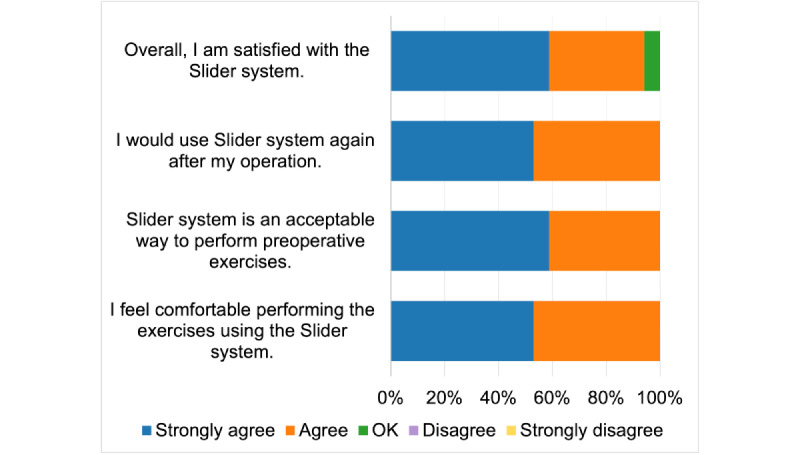
Telehealth Usability Questionnaire satisfaction and future use metric.

Figure 7 presents the results for TUQ’s satisfaction and future use metric. A total of 16 (94%) participants (10 participants strongly agreed and 6 participants agreed) agreed that they were satisfied with the device, whereas 1 (6%) participant stated they were OK with the system. None of the participants disagreed with the statement.

All the participants (9 strongly agreed and 8 agreed) stated that they would use the device again after the operation. None of the participants disagreed with the statement.

All participants (10 strongly agreed and 7 agreed) stated that the device was an acceptable way to perform preoperative exercises. None of the participants disagreed with the statement. All the participants (9 strongly agreed and 8 agreed) stated that they felt comfortable performing their exercises with the device. None of the participants disagreed with the statement. Overall, at least 16 (94%) participants were satisfied, and all agreed that they would use the device again.

**Table 2:** Demographics information of the participants.

Demographics		Participants (N=17), n (%)
Participants		17 (100)
Reported adverse effects		0 (0)
**Age distribution (years)**		
	55-64	3 (18)
	65-74	8 (47)
	75-84	6 (35)
**Sex**		
	Male	7 (41)
	Female	10 (59)
**Exercise side**		
	Right	10 (59)
	Left	7 (41)

## Discussion

### Principal Findings

This study set out to evaluate the usability and acceptability of Slider for at-home knee rehabilitation exercises. The results from 17 participants showed that a high percentage found the device useful, easy to learn and use, and satisfactory for their preoperative physiotherapy needs. Most participants agreed they would use the device again.

Prior research shows that enabling at-home rehabilitation can improve adherence and outcomes compared to traditional in-clinic therapy alone. For example, a randomized trial by Xu et al [11] compared standard in-clinic rehab to a home exercise program after total knee replacement. The home group used a self-developed program with a low stool to improve knee flexion. While both groups improved in pain and function, the home group had a better knee range of motion 1 month after the operation. The home program provided similar overall outcomes while saving patients time and costs of in-clinic therapy. This demonstrates the feasibility of effective home-based rehab protocols. Similarly, a meta-analysis found that technology-assisted rehabilitation programs for total joint replacements improved adherence and clinical outcomes such as pain and mobility [12]. Remote delivery provides convenience while tracking promotes accountability.

The quick learning curve found in this study is promising given that knee replacement patients are often older adults with limited technical experience. The simple sliding movement may support adoption better than solutions requiring strapping on devices or using complicated software. The gamification features such as tracking repetitions and scores seem motivating based on participants’ reported ease of use and intentions to continue using the device. This aligns with the behavior change theories showing feedback and rewards can drive adherence [13].

### Limitations

This initial usability study has several limitations that should be considered when interpreting the results. First, the study relied solely on the TUQ for evaluation. The TUQ contains positively toned questions that may introduce bias. Additional quantitative and qualitative metrics are needed to provide a more comprehensive assessment of usability. Second, the sample size of 17, although adequate for initial piloting, lacks statistical power for drawing definitive conclusions. Further research should be powered appropriately and include larger samples.

Third, the 2-week study duration does not reflect typical rehabilitation protocols that last 8-12 weeks. Longer-term evaluation is required to fully assess the device’s use. Fourth, detailed information on user errors and recovery processes was not captured. This data would provide greater insight into usability issues.

Finally, participants’ exercise backgrounds were not considered. Understanding how the device may benefit users with varying exercise experiences could strengthen future research. While promising, the study captured limited objective data on actual adherence or clinical impacts. Follow-up studies should compare Slider against standard rehab protocols measuring adherence through system data, clinical outcomes such as range of motion, patient-reported outcomes such as pain and function, and use factors such as hospital readmissions. This will better substantiate any impacts on recovery. Cost-effectiveness analyses are also needed to quantify the value of investing in this technology.

The initial findings from this study indicate that the device could potentially be a viable option for at-home rehabilitation, given its usability and acceptability among a small group of users. However, extensive research with a larger participant pool and extended observation periods is essential to fully understand its influence on patient adherence and recovery after surgery. Should subsequent studies confirm its effectiveness, this device may become a useful addition to current rehabilitation practices, addressing increasing demands. Continued investigation is necessary to determine whether it could become a self-managed rehabilitation option for patients.

### Conclusions

The findings from this usability study suggest that the participants found the device to be user-friendly and straightforward to learn. The participants reported satisfaction with the interface and indicated a likelihood to reuse the device. This study’s scope was to evaluate the usability and acceptability of the device for individuals preparing for knee replacement surgery to conduct their physiotherapy exercises at home. It is important to note that the study’s findings pertain only to the usability and acceptability aspects; they do not extend to clinical outcomes. While the results hint at the potential for the device to improve the outpatient care experience, more comprehensive research is needed to draw definitive conclusions.

## Appendix

Multimedia Appendix 1 Telehealth Usability Questionnaire.

## Abbreviations

IRAS: Integrated Research Application System
NHS: National Health Service
TUQ: Telehealth Usability Questionnaire
