# Supplementary material for: A Device for Prehabilitation of Total Knee Replacement Surgery (Slider): Usability Study
Source: JMIR Form Res. 2023 Dec 18;7:e48055. doi: 10.2196/48055 (PMC10758943; doi:10.2196/48055)
Supplement: Multimedia Appendix 1 [file formative_v7i1e48055_app1.pdf]

Table S1. Telehealth Usability Questionnaire.

**Participant ID**						
Age	45-54	55-64	65-74	75-84		85+
Sex	Male		Female		Other	
Joint side	Left			Right		
**Components**	**Factors**	**Strongly disagree**	**Disagree**	**OK**	**Agree**	**Strongly agree**
**Usefulness**						
1	Slider system improves my access to physiotherapy services					
2	Slider system saves me time traveling to a hospital or specialist clinic					
3	Slider system provides for my physiotherapy needs before my operation					
**Ease of use and learnability**						
1	It was simple to use this system					
2	It was easy to learn to use the system					
3	I believe I could become productive quickly using this system					
**Interface quality**						
1	The way I interact with this system is pleasant					
2	I like using the system					
3	The system is simple and easy to understand					
4	This system is able to do everything I would want it to be able to do					
**Reliability**						
1	I think the exercises I could perform using Slider are the same as in-clinic					
2	Whenever I made a mistake using the system, I could recover easily and quickly					
3	The system gave error messages that clearly told me how to fix problems					
**Satisfaction and future use**						
1	I feel comfortable performing the exercises using Slider					
2	Slider system is an acceptable way to perform preoperative exercises					
3	I would use Slider system again after my operation					
4	Overall, I am satisfied with Slider					
